# Upper Gastrointestinal Crohn’s Disease: Shedding Light on the Obscure L4 Classification Meaning

**DOI:** 10.3390/jcm14228260

**Published:** 2025-11-20

**Authors:** Francesca Lusetti, Cristina Bezzio, Alice De Bernardi, Michele Puricelli, Gianpiero Manes, Simone Saibeni

**Affiliations:** 1 IBD Unit, Gastroenterology Unit, Rho Hospital, ASST Rhodense, 20017 Rho, MI, Italy; flusetti@asst-rhodense.it (F.L.); aldebernardi@asst-rhodense.it (A.D.B.); michele.puricelli03@universitadipavia.it (M.P.); gmanes@asst-rhodense.it (G.M.); 2 IBD Center, IRCCS Humanitas Research Hospital, 20089 Rozzano, Milan, Italy; cristina.bezzio@hunimed.eu; 3 Department of Biomedical Sciences, Humanitas University, 20072 Pieve Emanuele, Italy

**Keywords:** cross-sectional imaging, disease location, Montreal classification, therapy, upper gastrointestinal Crohn’s disease

## Abstract

**Background/Objectives**: Upper gastrointestinal Crohn’s disease (UGI-CD) is inconsistently defined and often underrecognized, obscuring epidemiology, complicating diagnosis, and delaying tailored care. The absence of a unified, operational definition with segment-specific criteria hinders reproducibility and comparability across studies. **Methods**: We performed a narrative synthesis of adult and pediatric studies and major guidelines, examining definitions, prevalence, diagnosis, and management. Where possible, findings were mapped to upper GI segments (esophagus, stomach, duodenum, proximal small bowel). **Results**: Definitions of UGI-CD are heterogeneous. Prevalence varies widely and rises with systematic foregut assessment; isolated upper GI disease without ileocolonic involvement is rare. Diagnosis relies on esophagogastroduodenoscopy with biopsies, complemented by cross-sectional imaging and enteroscopy. Management should extend beyond standard ileocolonoscopy, with segment-tailored monitoring. Anti-TNF agents show the most consistent efficacy in esophagogastroduodenal disease, whereas benefits are attenuated in proximal small bowel involvement. For stricturing disease, endoscopic balloon dilation is suitable for short, non-penetrating strictures but often requires repetition; surgery is preferred for complex anatomy or failed dilation. A short summary of the article’s main findings is provided. **Conclusions**: UGI-CD remains poorly standardized across definition, epidemiology, and management. We propose a pragmatic diagnostic and monitoring pathway and highlight priorities for research: segment-based reporting, inclusion of upper GI-only cohorts, and validation of treatment targets aligned with treat-to-target care—steps essential to improve early recognition and patient outcomes.

## 1. Introduction

Crohn’s disease (CD) is a chronic, idiopathic inflammatory bowel disease (IBD), characterized by segmental, transmural inflammation that can affect any part of the digestive system from the mouth to the anus [1,2]. Although the ileum and colon remain the most frequently involved segments, disease may also extend to the upper GI tract—including the esophagus, stomach, duodenum, jejunum, and proximal ileum—classified as the L4 phenotype according to the Montreal classification [3,4]. This phenotype remains underrecognized in clinical practice and underrepresented in research, resulting in persistent gaps in epidemiological and therapeutic understanding [3,4].

Upper GI CD (UGI-CD) presents distinctive diagnostic and management challenges. Symptoms are often subtle, nonspecific, or entirely absent, leading to delayed recognition or misdiagnosis [5,6]. A comprehensive assessment usually requires complementary imaging beyond standard endoscopy, such as intestinal ultrasound (IUS), magnetic resonance enterography (MRE), computed tomography enterography (CTE), capsule endoscopy (VCE), or device-assisted enteroscopy (DAE) [4,7]. The disease course can be severe, with complications including gastric outlet obstruction, strictures, malnutrition and impaired quality of life [8,9]. Furthermore, the absence of standardized diagnostic criteria and inconsistent reporting across studies has generated wide variability in prevalence estimates and clinical outcomes, complicating comparison and evidence synthesis [5,6].

Despite these clinical implications, current therapeutic strategies remain largely extrapolated from trials in ileocolonic disease [8,9]. Over the past decade, several international guidelines have extensively addressed medical and endoscopic management for ileal, colonic, and ileocolonic phenotypes, yet none provide segment-specific recommendations or algorithms for L4 disease [1,2,10,11]. This omission underscores the limited representation of UGI involvement in pivotal randomized trials, where eligibility typically requires endoscopic activity confined to the ileum and colon [12].

This narrative review aims to provide an updated and comprehensive overview of UGI-CD, examining current definitions, epidemiology, differential diagnosis, and treatment strategies. By integrating recent data and highlighting existing knowledge gaps, our goal is to support a more precise understanding and management of this under-studied disease location.

## 2. Materials and Methods

We conducted a narrative review of upper gastrointestinal Crohn’s disease (UGI-CD; L4), searching PubMed, Embase, and Scopus, and reviewing major guidelines (ECCO, AGA, ESPGHAN) from inception to 31 August 2025. We used the following search terms: Crohn disease; upper gastrointestinal/upper GI; esophagus/oesophagus; stomach/gastric; duodenum/duodenal; jejunum/jejunal; proximal ileum; gastroduodenal; esophagogastroduodenal; L4; Montreal classification; and Paris classification. We also included abstracts from major international congresses. Three authors (F.L., C.B., S.S.) independently screened and reviewed all eligible articles and examined their references for additional studies. Given the narrative design of this review, quantitative pooling or imputation of missing data was not performed. Incomplete or missing outcomes were noted and discussed as potential sources of bias.

## 3. Relevant Sections

### 3.1. Definition and Classification: Limitations of Current Criteria

The definition of UGI-CD has evolved over time but remains inconsistent and poorly standardized, with important implications for both clinical care and research.

The Vienna classification (2000) represented the first structured attempt to categorize CD based on age at diagnosis, disease location, and behavior [13]. In this framework, L4 was defined as any involvement proximal to the terminal ileum, encompassing gastroduodenal as well as jejunal lesions. Importantly, locations were mutually exclusive: a patient could be labeled as L1 (ileal), L2 (colonic), L3 (ileocolonic), or L4 (upper GI), but not a combination [13]. Consequently, patients with both jejunal and ileal disease were classified as L3, which effectively obscured the presence of UGI-CD. This restriction led to a systematic underestimation of L4 disease in epidemiological reports and created a framework poorly suited to capture its coexistence with more distal locations [4].

The Montreal classification (2006) addressed this limitation by redefining L4 as a modifier rather than a standalone category [3]. Thus, proximal disease could be recorded in addition to other locations (e.g., L1 + L4, L3 + L4), allowing recognition of the frequent overlap between upper GI and distal lesions [3]. Moreover, according to the Montreal classification, disease location should be defined by endoscopic or radiological evidence, supported by histology when available [3]. This implies that nonspecific findings such as mild gastritis should not automatically be interpreted as Crohn’s disease involvement, a point frequently neglected in epidemiological reporting, contributing to variability in reported prevalence.

Beyond these formal statements, real-world reproducibility of Montreal classification remains limited. In a recent Five-Nations survey, 38 gastroenterologists classified 20 Crohn’s vignettes using Montreal classification: specialists achieved 58% agreement with an expert board versus 49% for fellows/early-career clinicians, with within-group agreement only moderate (Fleiss κ ≈ 0.52–0.53) [14]. Such variability may translate into heterogeneity in clinical decision-making and trial eligibility.

In pediatric populations, the Paris classification (2011) introduced a further subdivision of L4 into L4a (disease located proximal to the ligament of Treitz, i.e., esophagus, stomach, and duodenum) and L4b (disease between the ligament of Treitz and the proximal ileum) [15]. This distinction reflects the clinical and prognostic heterogeneity of proximal versus mid-small bowel disease: while esophagogastroduodenal involvement (L4a) may often present with inflammatory lesions, jejunal or proximal ileal involvement (L4b) is strongly associated with stricturing complications and worse long-term outcomes [16,17,18]. Despite its relevance, this subdivision has not been formally adopted in adult classifications.

A recent scoping review of Crohn’s disease guidelines by Yuan and colleagues (2024) underscored the persisting definitional ambiguity [5]. Among more than 1100 records screened, only a handful of guidelines explicitly mentioned upper GI disease, and none was dedicated exclusively to UGI-CD. In most cases, “upper GI involvement” was mentioned only in relation to endoscopic or histologic findings, without a precise anatomical definition and with no clarity on whether lesions of the jejunum should be considered part of this category. Only the joint ECCO–ESGAR 2013 diagnostic guideline explicitly defined the upper GI tract as the esophagus, stomach, and duodenum, while the role of the jejunum remained inconsistent across documents [19].

Accordingly, the recent multi-society ECCO–ESGAR–ESP–IBUS diagnostic guideline (Part 1) again highlights upper-GI involvement and recommends considering EGD in adults at the time of a new CD diagnosis [11].

Notably, the pediatric Paris subdivision (L4a/L4b) has not been carried forward into adult guidelines, leaving a gap between pediatric and adult disease frameworks [5].

Taken together, these inconsistencies underscore the absence of a unified definition for UGI-CD. While the transition from Vienna to Montreal and Paris reflects progress toward greater precision, current adult classifications remain insufficiently detailed as summarized in Table 1, which compares the evolution of definitions and criteria for L4 Crohn’s disease across major classification systems. A harmonized framework is still lacking and represents a key unmet need in both clinical practice and research.

### 3.2. Epidemiology: Insights from Meta-Analyses and Cohort Studies

The actual epidemiology of UGI-CD is difficult to establish, mainly because of heterogeneous definitions and variability in diagnostic assessment. As already mentioned, studies restrict upper GI involvement to the esophagus, stomach, and duodenum, whereas others also include the jejunum or proximal ileum [5]. Furthermore, in adult CD, the upper endoscopy is reserved for patients with suggestive symptoms and usually allows the visualization only up to the second portion of the duodenum [20,21]. Similarly, although major adults guidelines recommend baseline cross-sectional imaging at diagnosis, real-world implementation remains inconsistent across centers; recent multicentre audits and Europe-wide surveys highlight substantial variation in access and timing of magnetic resonance enterography (MRE), computed tomography enterography (CTE), and intestinal ultrasound (IUS) and persistent scheduling constraints [22,23,24].

As a result, a substantial proportion of cases may remain undetected, particularly among asymptomatic patients, and differences in definitions and diagnostic approaches contribute to the variability in reported prevalence.

**Figure 1:**
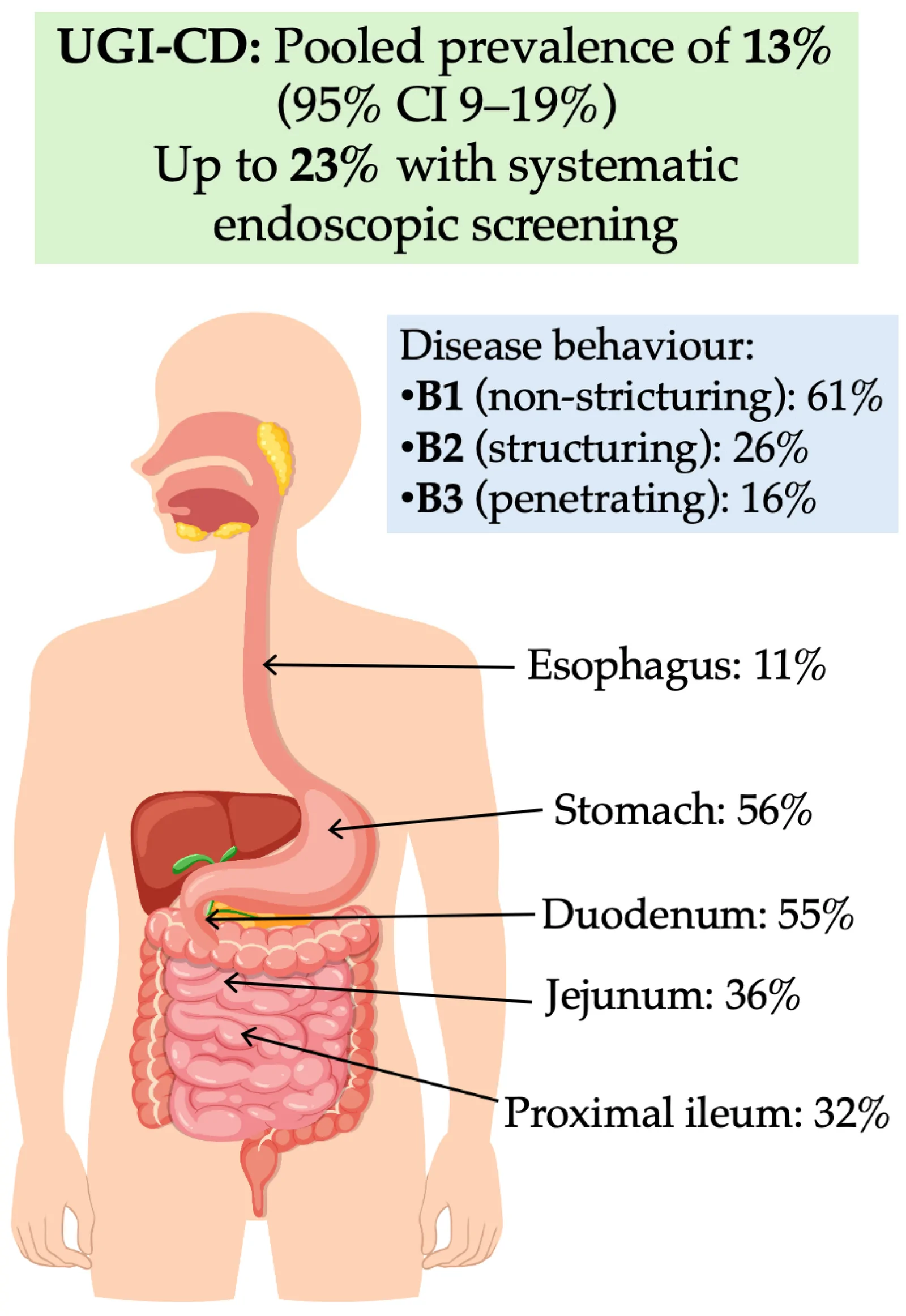
Prevalence, segment-specific distribution and disease behavior of UGI-CD [23].

A meta-analysis by Chin et al. (2021), which synthesized data from 26 studies involving more than 10,000 patients, reported a pooled prevalence of 13% (95% CI 9–19%) [25] (Figure 1). When analysis was limited to studies employing systematic endoscopic screening, prevalence increased to 23% (CI:13–35%; I2 = 90%) [25]. In terms of anatomical distribution, among patients with UGI-CD, the stomach (56%) and duodenum (55%) were the most frequently affected sites, while jejunal (36%) and proximal ileal (32%) involvement were also common; esophageal involvement was less frequent (11%) [25]. Regarding disease behavior, B1 (non-stricturing) was predominant (61%), followed by B2 (structuring, 26%) and B3 (penetrating, 16%) [18,25]. Importantly, as previously noted, jejunal involvement was associated with a higher likelihood of surgical intervention [18].

Subsequently, a systematic review by Cohen et al. (2025), which evaluated 47 studies including 6054 patients, found a median prevalence of 8.7% (IQR 4.7–24.4%) [26]. In cohorts where systematic upper endoscopy was performed, prevalence approached 30% [26]. Up to one-third of patients were asymptomatic, and among symptomatic individuals, abdominal pain was the most frequent complaint (41%). The duodenum was the most commonly affected site (69%), followed by the stomach (59%) and esophagus (16%).

Importantly, the systematic review by Cohen restricted UGI-CD to esophageal, gastric, and duodenal disease, without inclusion of jejunal or proximal ileal involvement. This definition, aligned with conventional upper endoscopy, likely contributes to the lower prevalence reported compared with studies that encompassed more distal segments of the proximal small bowel [26].

Findings from large cohort studies are consistent with these pooled estimates. In a North American cohort of 2105 patients, UGICD was identified in 8.3% of cases [18]. A Korean cohort of 811 patients reported a prevalence of 3%, predominantly involving the stomach and duodenum [27]. In the Dutch prospective cohort by Horje, 108 treatment-naïve patients with newly diagnosed CD underwent systematic upper endoscopy with biopsies: upper GI lesions were seen endoscopically in 55%, but only 41% fulfilled Montreal criteria for UGI-CD (compatible endoscopic features and/or histological confirmation); 68% of these were asymptomatic [28].

Crucially, across adult series UGI-CD rarely occurs in isolation: most patients are classified as L1/L2/L3 + L4 rather than L4 alone. Isolated L4 accounts for only 8–10%, whereas ileocolonic disease with L4 (L3 + L4) is the modal pattern [25,26,28].

### 3.3. Diagnosis and Differential Diagnosis: Challenges and Considerations

Accurate diagnosis of UGI-CD is intrinsically challenging. Clinical manifestations are frequently mild, non-specific, or absent, and no single modality provides a comprehensive evaluation of the proximal small bowel [26]. Symptoms, when present, cluster by segment yet rarely point unambiguously to UGICD: esophageal disease may cause pyrosis, odynophagia, or dysphagia; gastric/duodenal involvement typically presents with epigastric pain, dyspepsia, early satiety, nausea/vomiting, anemia or weight loss; jejunal/proximal ileal disease is enriched for post-prandial pain, vomiting, nutritional compromise and, in fibrostenosing phenotypes, obstructive symptoms [26,29].

On upper endoscopy, UGI-CD shows a recognizable—yet non-pathognomonic—constellation of findings that varies by segment and requires clinicopathologic correlation. In the esophagus, aphthous or longitudinal ulcers, friability, and occasionally strictures or fistulizing disease have been described, but no single feature is specific, and endoscopic appearances overlap with other esophagitis [26,30]. In the stomach, linear or serpiginous ulcers that track along rugal folds, nodularity, and the “bamboo-joint–like” pattern (raised intersecting ridges separated by furrows at the corpus/cardia) are characteristic clues reported in case–control and case series, although they may be absent in early disease [31,32]. In the duodenum, edema and erythema of folds, aphthae or longitudinal ulcers, cobblestoning, fixed narrowing and strictures are typical descriptions from classic and contemporary reports [26,33,34]. Histology increases specificity: non-caseating granulomas, when present, strongly support UGI-CD but are detected in a minority of biopsy series; focally enhanced gastritis (FEG) is frequently observed and supportive, particularly when *H. pylori* has been excluded, yet it is not diagnostic on its own—hence the emphasis on systematic biopsies, including from endoscopically normal mucosa [35,36].

Current multi-society guidance identifies ileocolonoscopy with segmental biopsies plus cross-sectional imaging (MRE, CTE, IUS) as the core of baseline diagnostic assessment in CD [2,37,38]. While ileocolonoscopy characterizes colonic and terminal ileal involvement, cross-sectional imaging delineates small-bowel extent and transmural complications (e.g., fistulas, abscesses, strictures, pre-stenotic dilatation). However, real-world access to CT/MR enterography remains uneven across centres and settings, as shown by the recent UK multi-centre audit by Taylor [23].

Interestingly, IUS provides a radiation-free, bedside evaluation of bowel-wall thickness, vascularity, and strictures, and—in many centers—is performed at the point of care by the treating gastroenterologist during the clinic visit, enabling rapid and repeatable, dynamic assessment, albeit with operator dependence [2,37,38].

In early, non-stricturing disease where inflammation may be confined to superficial mucosa and wall thickening is absent, video capsule endoscopy (VCE) can detect proximal small-bowel lesions and is suggested by recent studies and guidelines [39,40,41]. A recent network meta-analysis ranked VCE highest for diagnostic accuracy in small-bowel CD and especially for proximal involvement, compared to other modalities (pooled sensitivity of 89.6% for VCE, 82% for MRE, 79.6% for CTE and 89.3% for IUS) [42]. Capsule endoscopy carries a small risk of retention, and, in established CD, small-bowel patency should be assessed before VCE—using either a patency capsule or cross-sectional imaging. Data from meta-analysis show capsule retention rates of 2.4% in suspected and 4.6% in established CD, and these rates are 50% lower when small-bowel patency is confirmed [39].

Esophagogastroduodenoscopy (EGD), in adult patients, is suggested by major guidelines only when clinical or investigational cues suggest foregut disease. As previously mentioned, during EGD, gastric sampling should include testing for *H. pylori* to avoid misclassifying infection–related gastritis as UGICD, given that CD-associated focally enhanced gastritis is characteristically *H. pylori*–negative [36].

When jejunal or proximal ileal disease is suspected, cross-sectional imaging is preferred as the first step to detect mural inflammation and to stage strictures; if results are negative or equivocal and pre-test probability remains high, small-bowel capsule endoscopy is appropriate. Device-assisted enteroscopy (DAE) is reserved for targeted therapy or histology prompted by prior imaging/capsule findings. This multimodality sequence is consistent with ESGE guidance and is supported by head-to-head data showing that MRE, while valuable, has incomplete sensitivity for superficial mucosal lesions and for strictures compared with balloon-assisted enteroscopy [7,38,40].

A rigorous differential diagnosis is required prior to labelling UGI-CD [8,21,34]. Table 2 lists common L4 mimics, mapped to distinguishing features and suggested investigations.

### 3.4. Medical Management: Current Strategies and Gaps

Patients with an L4 phenotype remain under-represented—and frequently excluded—from therapeutic randomized clinical trials [43]. Indeed, across recent phase-3 programs for risankizumab (ADVANCE/MOTIVATE/FORTIFY), upadacitinib (U-EXCEL/U-EXCEED/U-ENDURE), and guselkumab (GALAXI), eligibility required ileocolonic endoscopic activity by SES-CD (typically ≥6; ≥4 for isolated ileitis). Consequently, patients with concomitant L4 involvement could be enrolled only if concomitant ileal/colonic disease was present, whereas L4-only phenotypes were effectively ineligible. Efficacy was then reported by the classical locations (ileal, colonic, ileocolonic), with no dedicated L4 subgroup analyses. This design perpetuates the evidence gap for UGI-CD and argues for segment-specific endpoints and prospective inclusion of L4-only disease in future trials [44,45,46,47].

#### 3.4.1. Anti-TNF Agents: Current Evidence and Segment-Specific Outcomes

In daily practice, we treat UGI-CD with the same advanced therapies used elsewhere in CD, but increasing evidence highlights site-specific differences in treatment response. A recent location-stratified meta-analysis of randomized controlled trials showed higher therapeutic efficacy in isolated colonic (OR 4.09 95% CI 3.02–5.54) than isolated ileal disease (OR, 1.80; 95% CI, 1.23–2.63; *p* < 0.001), confirming that treatment response varies by segment [48]. Similarly, in a complementary cross-sectional study using balloon-assisted enteroscopy, small-bowel remission under cytokine blockade (infliximab, adalimumab, ustekinumab) required higher serum drug concentrations than colonic disease, suggesting more extensive transmural inflammation in proximal segments [49].

Segment-specific analyses further reveal distinct response patterns across the upper gastrointestinal tract. For the foregut (esophagus, stomach, duodenum), evidence—though limited— supports anti-TNF benefit. The systematic review of adult UGI-CD by Cohen reported an overall clinical response of 81% with anti-TNF across available series [26]. Within this body of evidence, a multicentre series of 39 patients with esophageal CD showed higher clinical response (96.8% vs. 71.4%), clinical remission (64.5% vs. 14.2%), and endoscopic response (94.7% vs. 40.0%) with anti-TNF compared with other biologics (anti-integrin/anti-IL-12/23) [50]. In another prospective cohort, including gastroduodenal disease, endoscopic healing was achieved more frequently with anti-TNF than on mesalamine/thiopurines (72.7% vs. 12.5%) [29]. In the proximal small bowel, segment-specific data from BAE-based cohorts allow a more detailed assessment of therapeutic efficacy. In a multicentre prospective cohort (*n* = 253) with standardized BAE at baseline and week 26, endoscopic remission after biologic therapy (anti-TNF, ustekinumab, vedolizumab) was significantly less frequent in the proximal ileum than in the terminal ileum or colon; moreover, residual proximal ileal ulcers predicted hospitalization and surgery during follow-up [51]. Similarly, in the multicentre retrospective study by Huang et al., complete endoscopic healing at one year occurred in 21% overall—more frequent with infliximab (30%) than with ustekinumab (11%) or vedolizumab (6%). Healing was higher in the jejunum (31%) than in the proximal ileum (16%), confirming a gradient of response along the small bowel [52]. In a multicentre GETAID cohort of adults with UGICD strictures (predominantly duodenal in 60% of cases), anti-TNF therapy achieved 70% short-term clinical efficacy and was associated with a marked reduction in the need for surgery (odds ratio ≈0.2 at 1 and 5 years, whether used alone or with an immunomodulator) [53]. Real-world data from CREOLE study demonstrated that adalimumab yields short-term success in 2/3 of patients with symptomatic small-bowel strictures, with 50% surgery-free at 4 years in the overall cohort—highlighting that selected proximal small-bowel phenotypes can derive meaningful benefit but require careful selection and monitoring [54]. Together, these findings confirm that anti-TNF agents remain the cornerstone of therapy for L4 disease, although efficacy is clearly modulated by disease segment and phenotype.

#### 3.4.2. Beyond Anti-TNF Therapy: IL-12/23 Blockade, Integrin Antagonists, and Small Molecules

Evidence for non–anti-TNF biologics and small molecules in UGI-CD remains limited and derives almost entirely from small observational cohorts. Data suggest that their efficacy is substantially lower than that of anti-TNF agents, particularly in jejunal and proximal-ileal disease. Ustekinumab has shown only partial benefit. In the aforementioned multicentre study by Huang et al., endoscopic healing was achieved in 10–25% of patients with jejunal or proximal-ileal involvement—clearly inferior to infliximab (30%) in comparable populations [52]. In the multicentre GETAID cohort, clinical improvement occurred in 50% (1/2) on ustekinumab monotherapy and in 67% (2/3) when combined with an immunomodulator [53]. These findings, though limited by small sample size, suggest that IL-12/23 blockade may be modestly effective in inflammatory rather than fibrostenotic phenotypes.

Vedolizumab appears even less active in L4 disease. Across cohorts, rates of endoscopic healing ranged from 5–10%, significantly lower than with anti-TNF agents (endoscopic response 57.1% vs. 94.1%, *p* < 0.03; 21) [50,52]. The restricted efficacy of vedolizumab at this anatomical site likely reflects limited trafficking of α4β7-positive lymphocytes to the proximal small bowel.

Small molecules have also been evaluated only in exploratory settings. In the phase-2 DIVERGENCE-1 trial, which enrolled 78 patients with small-bowel Crohn’s disease (including 17 with jejunal involvement), filgotinib (100 mg or 200 mg) did not significantly improve transmural healing at week 24 compared with placebo. Jejunal healing occurred in 0% (0/8) with 100 mg, 33% (2/6) with 200 mg, and 0% (0/3) with placebo, although interpretation was limited by early discontinuation and small numbers [55].

Taken together, current data support a treat-to-target strategy that acknowledges attenuated healing rates in the proximal small bowel, positions anti-TNF agents as the most substantiated class for UGI-CD, and recognizes the modest benefit of other biologics or small molecules. Future studies should incorporate segment-stratified endpoints and dedicated L4 subgroups to clarify therapeutic efficacy across the proximal small bowel [25,26].

### 3.5. Endoscopic and Surgical Management for UGI-CD Strictures

As previously mentioned, UGI-CD is a difficult-to-treat phenotype, with a high burden of stricturing and a frequent need for surgical management, particularly in case of jejunal or proximal ileal involvement. In a 2105-patient consortium, L4-jejunal disease independently increased stricturing risk (OR 2.90, 95% CI 1.89–4.45) and multiple abdominal surgeries (OR 2.39, 1.36–4.20), exceeding risks in non-L4 ileal disease; stricturing was also associated with ileal location alone (OR 3.18, 2.23–4.64) and longer disease duration (OR 1.33 per decade) [18]. A single-centre cohort separating L4 subphenotypes showed higher surgery rates in L4 versus non-L4 during 5.8 years of follow-up (31% vs. 16%); risk was greatest for L4-jejunal (66%) and intermediate for L4-proximal ileal (28%), compared with L4-EGD (9%), with multivariable hazards of resection for L4-jejunal 3.08 (1.30–7.31) and L4-proximal ileal 1.83 (1.07–3.15) [16].

Interventional evidence for UGI-CD strictures is limited: no randomized trials compare endoscopic and surgical strategies in the foregut and available studies show considerable heterogeneity in treatments, patient populations, and outcome [3,25,36].

For short, accessible, non-penetrating strictures, endoscopic balloon dilation (EBD) is an appropriate first-line option, with high technical success, moderate short-term clinical efficacy (70–80%), low complication rates (2–3%), but a high need for repeat dilations over time; technique should follow pragmatic standards (pre-procedural cross-sectional imaging; ≤3 graded inflations, 60–90 s each; target diameter 15–18 mm) and should be avoided in the presence of deep ulceration or suspected malignancy [51].

Consistently, some longer-term EBD series regarding duodenal disease report high technical success (98.69%) with frequent need for re-dilation over time [52], and broader benign-duodenal stenosis cohorts outline practical technique and outcomes supporting EBD as a surgery-sparing option in short, accessible, non-penetrating strictures, regardless of etiology [53].

A retrospective comparative study on duodenal strictures specifically showed that surgery achieved greater symptomatic improvement (100% vs. 63.3%) and a significantly longer recurrence-free survival (median 6.31 [IQR 3.00–8.39] years) than EBD (2.96 [1.06–5.42] years), albeit with more post-procedural adverse events (16.7% vs. 0.74% per procedure); among those initially managed with EBD, 27% ultimately required surgery [54].

However, selection remains crucial: longer (>5 cm), angulated, or multiply segmented strictures—and any with penetrating complications—are poor candidates for durable endoscopic control and should prompt surgical planning [15,54,55,56].

Contemporary foregut surgery for CD favors bowel-preserving approaches where possible—strictureplasty (Heineke–Mikulicz/Finney) for short/multiple lesions and bypass (gastrojejunostomy or duodenojejunostomy) for complex gastric outlet/duodenal disease—reserving segmental resection for selected scenarios [49,57,58].

Collectively, the evidence supports a stepwise, anatomy-guided strategy, firstly defining location, length, and complications with cross-sectional imaging. In short, for accessible, non-penetrating foregut strictures, EBD using standardized, graded inflations is appropriate and should be coupled with optimization of anti-inflammatory therapy. Surgery is preferred for long, angulated, or multifocal disease, for penetrating complications, or after relapse despite technically adequate dilatation. Postoperative surveillance should be planned from the outset.

## 4. Discussion

The involvement of upper gastrointestinal tract remains a rare and underexplored phenotype of Crohn’s disease. The scarcity of dedicated trials, coupled with inconsistent diagnostic definitions and indications as well as the frequent exclusion of L4 patients from pivotal studies, has resulted in a fragmented evidence.

This phenotype is inconsistently defined and underrecognized in clinical practice; the absence of an operational, adult framework (e.g., L4a/L4b) limits comparability across studies and impedes appropriate care. Epidemiologic estimates are heterogeneous because many patients are asymptomatic, and routine assessments rarely interrogate the foregut and proximal small bowel comprehensively [22,23]. Diagnosis should integrate cross-sectional imaging (MRE/CTE/IUS), selective EGD with systematic sampling including *H. pylori* testing, and enteroscopy (VCE after patency when proximal small-bowel disease is suspected and DAE with biopsies). Recent diagnostic consensus statements emphasize the need for multimodal evaluation combining endoscopy, imaging, and histology, given that isolated proximal lesions may otherwise be missed [11,39].

Therapeutic evidence is sparse owing to the frequent exclusion of UGI-CD patients from trials; when included, outcomes are generally not analysed or reported by L4 status or sublocation. Most available data derive from retrospective or registry-based cohorts aggregated in meta-analyses, highlighting an almost complete absence of randomized, segment-specific evidence [4,25,48]. The heterogeneity and retrospective nature of these studies inevitably introduce bias, limiting the strength and generalizability of current findings. Consequently, the conclusions of this review should be interpreted as hypothesis-generating, pending confirmation from prospective, segment-focused trials. These analyses collectively indicate that anti-TNF agents achieve the highest rates of clinical and endoscopic response across upper-GI segments, whereas IL-12/23 blockade, integrin antagonists, and small molecules demonstrate substantially lower efficacy—particularly in jejunal and proximal-ileal disease [52,53,54]. Available data support anti-TNF as the most substantiated option for foregut disease, while treatment effects in the proximal small bowel—especially the proximal ileum—appear attenuated. This gradient of efficacy, confirmed in both endoscopic and imaging-based cohorts, likely reflects the greater depth of transmural inflammation and altered pharmacokinetics in proximal segments, which may require higher drug exposure to achieve comparable healing [51].

UGI-CD often presents as a complicated, predominantly stricturing phenotype, with a substantial need for endoscopic or surgical management. In the GETAID and CREOLE cohorts, early anti-TNF therapy was associated with significant reductions in surgery risk (odds ratio ≈ 0.2–0.25), supporting its use as first-line advanced therapy even in fibrostenotic presentations [53,54]. In these cases, endoscopic balloon dilation (EBD) provides safe, repeatable relief for carefully selected short, nonpenetrating strictures, whereas surgery affords more durable patency in complex disease. EBD achieves technical success in >95% of cases and short-term clinical response in 70–80%, though recurrence remains common [59,60,61].

Overall, the body of evidence supports a pragmatic, segment-oriented approach: anti-TNF agents as the cornerstone of therapy, ustekinumab reserved for inflammatory phenotypes refractory to anti-TNF, and vedolizumab or small molecules considered on a case-by-case basis. The paucity of comparative data precludes clear sequencing or combination strategies, reinforcing the need for individualized management guided by disease behaviour, segmental location, and therapeutic drug monitoring [25,26,52].

Future studies should broaden eligibility to include L4-only and L4-predominant disease, report outcomes by upper-GI segments, and validate UGI-specific endoscopic and imaging targets to enable treat-to-target strategies [12,24,43]. Priority should be given to ad hoc randomized controlled trials tailored to the L4 location—particularly jejunal and proximal ileal disease—across both medical therapy and stricture management. For medical therapy, trials should stratify by segment (EGD, jejunal, proximal ileal) and behavior, prespecify segment-level endpoints (small-bowel mucosal healing on device-assisted enteroscopy and transmural remission on MRE), and incorporate pharmacokinetic–pharmacodynamic targets with therapeutic drug monitoring. For procedural care, comparative RCTs should evaluate EBD strategies versus early surgery for short primary proximal strictures, and surgery versus optimized medical therapy for fibrostenotic disease, using core outcomes such as time to reintervention, durable patency, need for resection or stoma, nutritional status, and patient-reported outcomes.

## 5. Conclusions

UGI-CD remains a poorly standardized and frequently overlooked manifestation of Crohn’s disease, with major diagnostic and therapeutic gaps.

Current data—derived mainly from retrospective or observational studies—indicate that anti-TNF agents provide the most consistent therapeutic benefit, with clinical response rates of 70–85% and endoscopic healing in 30–50% of cases, particularly for esophageal and gastroduodenal disease. In contrast, ustekinumab, vedolizumab and small molecules demonstrate limited efficacy, achieving ≤25% and ≤10% mucosal healing, respectively.

Treatment outcomes in the proximal small bowel remain suboptimal, with jejunal healing around 30% and proximal ileal healing at 10–15%, suggesting that disease location and phenotype markedly influence therapeutic response.

Given that fibrostenotic complications are frequent, EBD could represent an effective, minimally invasive option for short, non-penetrating strictures, while surgery provides durable results in complex disease. New drugs with direct anti-fibrotic potential are currently under investigation and could be used in this peculiar phenotype to prevent, reduce or reverse fibrosis by targeting specific pathways.

Future research should focus on segment-specific clinical trials that include L4-predominant disease and define validated endpoints such as device-assisted enteroscopy-confirmed mucosal healing and transmural remission on MRE. Establishing standardized definitions, diagnostic criteria and outcome measures will be essential to improve comparability and guide individualized management strategies for UGI-CD.

## Figures and Tables

**Table 1:** Evolution of Upper GI Crohn’s Disease Classification.

Classification System	Definition of L4	Target Population	Advantages	Limitations
**Vienna (2000)**	Any disease located **proximal to the terminal ileum**, including gastroduodenal, jejunal, or proximal ileal involvement. Locations were mutually exclusive (L1 − L4).	Adults	First structured classification to include disease location and behavior; introduced concept of “upper GI” Crohn’s disease.	Proximal and distal locations could not coexist (e.g., L3 + L4 not allowed); led to underestimation of L4 prevalence.
**Montreal (2006)**	Redefined L4 as a **modifier** that can be added to L1, L2, or L3 when proximal disease is present (e.g., L1 + L4, L3 + L4).	Adults	Allowed coexistence of proximal and distal disease; improved standardization using endoscopic/radiologic confirmation.	Anatomical boundaries not precisely defined (unclear inclusion of jejunum); only moderate interobserver reproducibility.
**Paris (2011)**	Subdivided L4 into **L4a** (esophagus, stomach, duodenum—proximal to the ligament of Treitz) and **L4b** (jejunum and proximal ileum—distal to the ligament of Treitz).	Pediatric	Highlighted clinical and prognostic differences between foregut and mid–small bowel disease; greater phenotypic granularity.	Limited to pediatric populations; not yet adopted in adult classification; contributes to discontinuity between age groups.

Abbreviations: L4, upper gastrointestinal Crohn’s disease; GI, gastrointestinal.

**Table 2:** Main differential diagnoses of upper gastrointestinal Crohn’s disease and distinguishing features.

L4 Site	Main Differential Diagnoses	Key Distinguishing Features and Tests
**Esophagus**	Infectious esophagitis (*HSV, CMV, Candida* spp.); Eosinophilic esophagitis (EoE)	Immunosuppression, punched-out ulcers, viral inclusions (PCR/IHC); rings/furrows, ≥15 eosinophils/HPF, atopy, response to topical steroids or PPIs
**Stomach/** **Duodenum**	*H. pylori* gastritis; *CMV/HSV* infection; NSAID gastroduodenopathy; Eosinophilic gastroenteritis; Celiac disease; Sarcoidosis; Behçet disease	Positive *H. pylori* tests; viral inclusions; NSAID use with diaphragm-like webs; eosinophilia; celiac serology (tTG-IgA/EMA+, HLA-DQ2/DQ8); systemic granulomatous or vasculitis features
**Jejunum/** **Proximal ileum**	Intestinal tuberculosis; NSAID enteropathy; Small-bowel lymphoma, adenocarcinoma, NET, GIST; CVID enteropathy; CMUSE; CEAS	Caseating granulomas or AFB+; multiple short strictures, minimal inflammation; mass lesions with clonal markers (e.g., *c-KIT*, chromogranin); low immunoglobulins; poor response to immunosuppression; *SLCO2A1* variants in CEAS
**Any L4 Site**	Vasculitis/Ischemic enteritis	Systemic vasculitis signs (rash, arthralgia, renal/neurologic involvement); vessel-centered inflammation; ischemic injury pattern

Abbreviations: AFB, acid-fast bacilli; CEAS, chronic enteropathy associated with *SLCO2A1*; CMUSE, cryptogenic multifocal ulcerous stenosing enteritis; CVID, common variable immunodeficiency; EMA, anti-endomysial antibody; GIST, gastrointestinal stromal tumor; IHC, immunohistochemistry; NET, neuroendocrine tumor; PCR, polymerase chain reaction; PPI, proton pump inhibitor; tTG, anti-tissue transglutaminase.

## Data Availability

Data sharing is not applicable to this article as no new data were created or analyzed in this study.
